# No Association between Mean Telomere Length and Life Stress Observed in a 30 Year Birth Cohort

**DOI:** 10.1371/journal.pone.0097102

**Published:** 2014-05-09

**Authors:** Sarah Jodczyk, David M. Fergusson, L. John Horwood, John F. Pearson, Martin A. Kennedy

**Affiliations:** 1 Gene Structure and Function Laboratory, Department of Pathology, University of Otago, Christchurch, New Zealand; 2 Christchurch Health and Development Study, Department of Psychological Medicine, University of Otago, Christchurch, New Zealand; 3 Department of the Dean, University of Otago, Christchurch, New Zealand; University of Otago, New Zealand

## Abstract

Telomeres are specialised structures that cap the ends of chromosomes. They shorten with each cell division and have been proposed as a marker of cellular aging. Previous studies suggest that early life stressors increase the rate of telomere shortening with potential impact on disease states and mortality later in life. This study examined the associations between telomere length and exposure to a number of stressors that arise during development from the antenatal/perinatal period through to young adulthood. Participants were from the Christchurch Health and Development Study (CHDS), a New Zealand longitudinal birth cohort which has followed participants from birth until age 30. Telomere length was obtained on DNA from peripheral blood samples collected from consenting participants (n = 677) at age 28–30, using a quantitative PCR assay. These data were assessed for associations with 26 measures of life course adversity or stress which occurred prior to 25 years of age. No associations were found between telomere length measured at age 28–30 years and life course adversity or stress for specific measures and for the summary risk scores for each developmental domain. The correlations were very small ranging from −0.06 to 0.06 with a median of 0.01, and none were statistically significant. Our results in this well-studied birth cohort do not support prior reports of such associations, and underscore the need for more extensive replication of proposed links between stress and telomere biology in larger cohorts with appropriate phenotypic data.

## Introduction

Telomeres are complex structures made up of nucleic acid and protein, located at the ends of chromosomes where they provide a protective cap and maintain the integrity of the genome [1], [2]. Telomeric DNA is gradually lost each time a cell divides, until the length of any given telomere reaches a critical level that triggers cell senescence and death [1]. Average telomere length measured in peripheral blood cells decreases as humans age, and telomere length erosion may be a potentially useful biomarker of lifetime exposure to environmental and biological stressors [3]–[5].

Telomere length is influenced by the cumulative effect of genetic factors [6]–[9] and the ‘end replication problem’ where successive cycles of cell division progressively shorten telomeric DNA [1]. The enzyme telomerase has the ability to extend telomere length, but telomerase is only up-regulated in certain highly proliferating cells including germ cells, and down-regulated in most other tissues of the body [10], [11]. The recognition that telomere length is determined by dynamic processes has given rise to many studies examining links between average telomere length and various stressors, with mixed findings. Some studies have found that overall physical health or specific environmental stressors such as smoking [12]–[15] are associated with altered telomere length, whereas others have not [16]–[18]. Epel *et al* (2004) made the association between self-perceived psychological stress and the reduction in telomere length due to the prolonged exposure to a highly stressful situation brought on by care-giving for a chronically ill child [4], which has been further supported by subsequent studies [19], [20].

In addition to perceived stress in adulthood, there is now a growing body of evidence that stress during fetal development [21], [22] or as a child, including sexual or emotional abuse, violence or other adverse events, also leads to shortened telomeres in adulthood [23]–[29], although not all studies concur [30]. Early life stress can also predispose to mood disorders or mental illness, and some of these, including schizophrenia [31] and depression [32], [33], have been associated with shorter telomeres, although again, findings are not consistent [34].

Research in this area has suffered a number of limitations. The most important of these has been that most studies have used retrospective report measures of stress and adversity. It is possible that these measures are affected by a reporting bias in which those experiencing accelerated aging are more likely to report life course adversity. A closely related problem is that few studies have used a life course perspective that examines the effects of stressful exposures at various stages of human development. However, one recent example of a longitudinal study found that exposure to abuse during childhood was associated with a significant erosion of telomere length [27].

The Christchurch Health and Development Study (CHDS) is a longitudinal study of a New Zealand birth cohort from birth to young adulthood with telomere length measured at age 28–30. The measures of life course stress included: (1) antenatal/perinatal events including maternal smoking during pregnancy, low birth weight, being small for gestational age, admission to neonatal intensive care; (2) exposure to adverse childhood events including childhood physical and sexual abuse, neglect, inter-parental conflict and violence, and related sources of childhood adversity; (3) exposure to adverse events in adolescence and young adulthood, including drug and alcohol behaviours, mental health, lifetime trauma and major events, and pregnancy/parenthood events. We aim to identify and quantify associations between these measures of life course adversity and average leukocyte telomere length (LTL).

On the basis of previous research and theory it was hypothesized that:

1. LTL at age 28–30 would be related to the individual's exposure to adverse antenatal/perinatal, childhood, adolescent and adult experiences.
2. The reduction in LTL would be proportional to the individual's cumulative exposure to life course adversities.

More generally the aims of this paper are to take a life-course perspective by examining the ways in which a number of biological, social and other stressors are related to telomere length in young adulthood.

## Materials and Methods

### Ethics statement

All phases of the study were subject to ethical approval from the Canterbury Regional Health and Disability Ethics Committee (New Zealand). Collection of blood for extensive DNA analysis and all forms of data collection were subject to the informed, written consent of study participants.

### Subjects

The data were gathered during the course of the Christchurch Health and Development Study (CHDS). This study is a birth cohort of 1265 children born in the Christchurch (New Zealand) urban region in mid-1977 with data collection carried out at birth, 4 months, 1 year and annually to age 16 years, and again at ages 18, 21, 25 and 30 years [35], [36]. Sample retention rates were high throughout the study and at age 30 the study was still able to assess over 80% of the surviving cohort. The present analysis is based on a sample of 677 cohort members (86% white, 14% NZ Maori/Pacific Island ethnicity) who were assessed on measures of life course stress or adversity up to age 25 and for whom mean LTL measures were successfully obtained.

### Telomere length measurement

DNA was extracted from peripheral blood using a sodium chloride precipitation procedure [37]. 916 CHDS participants at age 28–30 consented to provide a peripheral blood or saliva sample for DNA analysis. Once poor quality DNA samples and those derived from saliva were excluded, we were left with 739 good quality DNA samples available for this analysis. Of these, 677 gave acceptable telomere length measures which met our quality control criteria (described below).

Average LTL was determined using a quantitative polymerase chain reaction (qPCR) method [38], [39] with several modifications as described in Jodczyk *et al*, (manuscript submitted). This method provides a relative measure of average telomere length from genomic DNA expressed as a T/S ratio by measuring the number of telomere repeats (T) across all chromosomes for all cells relative to the amount of a single copy reference gene (S). The qPCR reactions for both the telomere and single copy reference gene were performed separately in duplicate on the Roche LightCycler 480 real-time PCR machine (Roche, Mannheim, Germany), ensuring that the T and S reactions for each sample were located in the same relative well position in a 384 well format. An average of the duplicate measures was used to calculate the T/S ratio by dividing the telomere product by the single copy reference gene product.

The primers for the telomere PCR were telg (5′ACACTAAGGTTTGGGTTTGGG TTTGGGTTTGGGTTAGTGT3′) and telc (5′TGTTAGGTATCCCTATCCCTATC CCTATCCCTATCCCTAACA3′) used at a final concentration of 900 nM. The primers for the single copy reference gene (Albumin) PCR were albu (5′CGGCGGCGGGCGGCGCGGGCTGGGCGGAAATGCTGCACAGAATCCTTG3′) and albd (5′GCCCGGCCCGCCGCGCCCGTCCCGCCGGAAAAGCATGGTCGCCTGTT3′) [39] used at a final concentration of 900 nM. The thermal cycling profile for the telomere PCR consisted of the following steps. Stage 1: 95°C for 15minutes; Stage 2: two cycles of 94°C for 15 s, 49°C for 15 s; Stage 3: 35 cycles of 94°C for 15 s, 62°C for 10 s, 74°C for 15 sec with signal acquisition. The profile for the albumin gene PCR consisted of the following steps. Stage 1: 95°C for 15minutes; Stage 2: two cycles of 94°C for 15 s, 49°C for 15; Stage 3: 40 cycles of 94°C for 15 s, 62°C for 10 s, 88°C for 15 sec with signal acquisition.

Sample concentration was determined using the standard curve method prepared by two fold serial dilutions of a reference genomic DNA sample ranging from 1.56–100 ng. LightCycler 480 software 1.5.0 (Roche, Mannheim, Germany) was used to convert the cycle threshold (C_t_) to nanograms of DNA using the second derivative method.

The main analysis is based on samples with two acceptable replicates (N = 677) which were determined using the criterion that if the coefficient of variation was greater than 10% for the replicates then the result was disregarded, and the sample was re-assayed. This equated to approximately 10% of the cohort. In addition to this, each assay included three quality control samples of varying telomere length (short, medium and long) which gave an average inter-assay coefficient of variation for telomere length measurement of 7% for this study. To confirm that the assay was performing adequately 20 control DNA samples were assayed with both this T/S method and the Terminal Restriction Fragment (TRF) Southern blot method [40] and found to give an overall correlation (r^2^ = 71, or r = 0.853, equivalent to r = 0.847 reported by Aviv and Blackburn [41] (Jodczyk *et al*, manuscript submitted).

**Figure 1:**
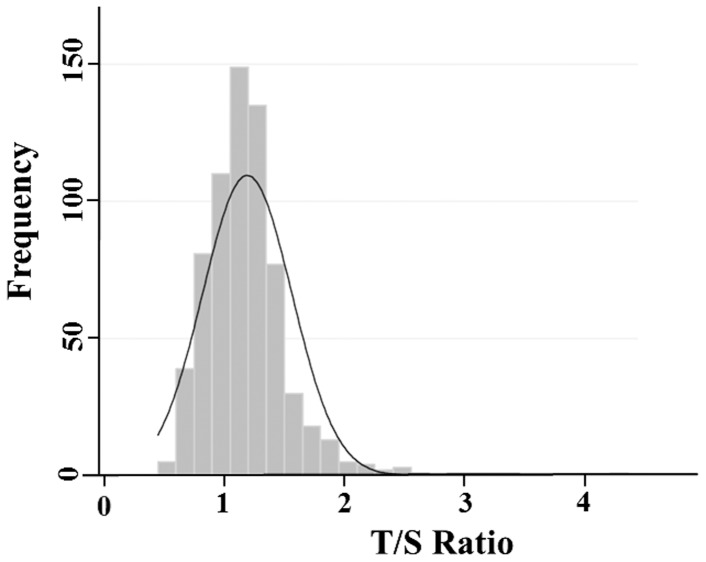
Distribution of LTL. The distribution of observed telomere lengths (T/S ratio) for the CHDS cohort (n = 677) is shown as a histogram, a density curve for a normal distribution with the same mean (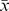 = 1.18) and standard deviation (s = 0.37) has been superimposed. The data is not normally distributed (Shapiro-Wilks p<0.001), and is right skewed (skewness 2.3). Telomere length in this cohort ranges from a T/S ratio of 0.50 to 4.35.

Figure 1 shows the distribution of LTL in the sample. The distribution was leptokurtic (thinner) and skewed to the right in comparison to a normal distribution (skewness = 2.3; kurtosis 12.3, Shapiro-Wilk test of normality p<.001).

### Measures of life course stress or adversity (0–25 years)

The following measures were selected from the database of the study to characterise exposure to various forms of stress and adversity over the life course. With one exception (see below), all measures were assessed prior to the collection of DNA and assessment of LTL. (See online supporting information Table S1 for summary data on these measures). The measures spanned the following domains:

#### Antenatal/perinatal factors

Selected factors included child birth weight, gestation (weeks since last menstrual period), admission to neonatal intensive care (no/yes) following birth, and maternal smoking (cigs/day) during pregnancy. To provide an overall measure of antenatal/perinatal adversity a risk score was constructed from a sum of four dichotomous indicators reflecting potential risk/adversity on each of the above measures. These indicators were: child of low birth weight (<2500 g), child born premature (<36 weeks gestation), admission to neonatal intensive care, and mother smoked a pack a day or more during pregnancy.

#### Child abuse/family violence (0–16 years)

Exposure to abuse, violence and neglect during childhood (<16 years) was assessed in the following ways. Child sexual abuse (CSA) exposure was assessed using retrospective reports obtained at ages 18 and 21 years for a range of unwanted sexual experiences in childhood. Using these data participants were classified on a 4-level scale reflecting the most severe form of abuse reported at either age: no CSA; non-contact abuse only; contact abuse involving inappropriate touching, fondling of genitals; attempted or completed sexual penetration [42], [43]. Child physical abuse (CPA) was assessed on the basis of retrospective reports obtained at ages 18 and 21 of the extent to which the individual's parents used physical punishment during their childhood. Using these data, participants were classified on a 4-level scale reflecting the most severe form of punishment reported at either age: parents never used physical punishment; parents rarely used physical punishment; at least one parent regularly used physical punishment; at least one parent used severe or harsh physical punishment [42], [43]. At age 16 participants completed the parental care scale of the Parental Bonding Instrument [44]. This scale assesses the extent to which the parents are reported to have provided a loving, caring and supportive home environment during childhood. Separate ratings were obtained for mothers and fathers and these ratings were averaged to provide an overall assessment of the level of care [45]. Exposure to inter-parental violence and conflict was assessed on the basis of retrospective reports obtained at age 18. Participants were questioned using a selected series of items from the Conflict Tactics Scale [46] to assess the extent to which they reported witnessing incidents of inter-parental violence and conflict during childhood. These items were combined to produce an overall scale measure of inter-parental violence/conflict [47]. Finally, to provide an overall measure of exposure for child abuse/family violence a risk score was constructed from a sum of four dichotomous indicators reflecting exposure to different forms of abuse, violence or neglect. These indicators were: child exposed to CSA involving inappropriate contact or attempted/completed intercourse; child exposed to regular or severe physical punishment; child fell into the lowest decile on the measure of parental care; child fell into the highest decile on the measure of interparental violence/conflict.

#### Adolescent/young adult substance misuse (16–25 years)

At ages 18, 21 and 25 years participants were interviewed about their use/misuse of tobacco, alcohol, cannabis and other illicit drugs over the period since the previous assessment [42]. As part of this interview, items from the Composite International Diagnostic Interview (CIDI) [48] were used to assess DSM-IV [49] symptom criteria for alcohol, cannabis and other drug dependence; symptoms of nicotine dependence were assessed on the basis of custom written survey items. These symptom data were combined over the three interview periods to construct dichotomous measures reflecting whether the participant had ever met DSM-IV diagnostic criteria for nicotine dependence, alcohol dependence, cannabis dependence and other drug dependence over the period from age 16–25 years. An overall measure of extent of exposure to problem substance use behaviours was also constructed based on a count of the number of substance use disorders for which the individual met diagnostic criteria.

#### Adolescent/young adult mental health (16–25 years)

As part of the interviews conducted at ages 18, 21 and 25 participants were questioned using components of the CIDI to assess DSM-IV symptom criteria for major depression and a range of anxiety disorders (generalised anxiety disorder, panic disorder, agoraphobia, social phobia, specific phobia). Participants were also questioned about suicidal behaviours including suicidal ideation and suicide attempt since the previous assessment [42]. These data were used to classify participants on three dichotomous measures of mental health problems reflecting whether the participant met DSM-IV diagnostic criteria for major depression, or an anxiety disorder, or reported suicidal ideation/attempt over the period from age 16–25 years. In addition at age 30, as part of questioning about symptoms of PTSD, participants were questioned about their lifetime exposure to a series of potentially traumatic life events [50]. A measure of total lifetime exposure to trauma was constructed from a count of the number of traumatic events reported. Finally, an overall measure of mental health problems was constructed by summing the three dichotomous measures of depression, anxiety disorder and suicidal ideation/attempt together with a four dichotomous measure reflecting whether the participant fell into the highest quartile of exposure to lifetime trauma.

#### Adolescent/young adult life events (16–25 years)

As part of the interviews conducted at ages 18, 21 and 25 participants were questioned about life events occurring for each 12 month period since the previous assessment using a life events checklist that combined custom written items with selected items from existing life event scales [51]. These data were used to classify participants on five broad measures reflecting common classes of life event. These classes were: employment related events (redundancy, unemployment, changes of job); serious illness or accident to self, close friends or family members; serious relationship problems with friends, partners or other family members; victimisation events (physical/sexual assault, robbery, etc); and pregnancy/parenthood events (pregnancy/getting a partner pregnant, pregnancy loss, termination, childbirth). For each class of event a score was constructed based on a count of the total number of events reported over the period 16–25 years. Finally, a measure of total life event exposure was constructed by first summing all life events reported for each year from age 16–25 and then averaging over all years to provide a measure of the average number of life events reported over the whole interval.

### Statistical analysis

In preliminary analysis one way analysis of variance was used to test for differences in mean LTL by sex, ethnicity (European, Maori/Pacific Island) and family socio-economic status at the participant's birth (classified into three levels on the basis of paternal occupation: professional/managerial; clerical/technical/skilled; semi-skilled/unskilled). LTL was unrelated to sex or SES. However, Maori/Pacific Island participants had significantly higher mean LTL than Europeans (p = .04). To control for possible population stratification, the associations between LTL and the measures of life course stress/adversity (Table 1) were examined by fitting a series of multiple linear regression models in which LTL was regressed on each measure of stress/adversity, controlling for sex, ethnicity and family SES at birth. For each stress measure the strength of the adjusted association with LTL was estimated by the partial correlation (standardised regression coefficient) and corresponding test of significance from the fitted model. Application of standard power calculations [52] showed that with 677 cases, the sample had in excess of 80% power at α_2_ = .05 to detect a partial correlation in excess of .11. This suggests that the sample had adequate power to detect small effect sizes.

**Table 1:** Standardised regression coefficients (β) linking measures of developmental stressors (birth – 25 years) and average leukocyte telomere length (28–30 years) adjusted for sex, ethnic origin and family SES at birth (N = 677).

Measure	Standardised β	Measure	Standardised β
Antenatal/Perinatal Factors		Adolescent/Young Adult Mental Health (16–25 years)	
Birth weight (g)	−.01	DSM-IV Major depression	.03
Gestation (wk)	−.05	DSM-IV Anxiety disorder	.02
Admission to neonatal intensive care	.01	Suicidal ideation/attempt	−.01
Maternal smoking in pregnancy (cigs/day)	−.02	Lifetime trauma	−.01
Perinatal risk score	.04	Total mental health problems	.03
Child Abuse/Family Violence (0–16 years)		Adolescent/Young Adult Life Events (16–25 years)	
Childhood sexual abuse	.06	Employment problems	−.02
Childhood physical abuse	.03	Serious illness/accident to self or others	−.01
Parental care	−.06	Serious relationship problems	.01
Inter-parental violence/conflict	−.01	Victimization problems	−.01
Abuse/violence risk score	.05	Pregnancy/parenthood	.04
Adolescent/Young Adult Substance Use (16–25 years)		Total life events exposure	−.02
DSM-IV Nicotine dependence	.01		
DSM-IV Alcohol dependence	.01		
DSM-IV Cannabis dependence	.02		
DSM-IV Other drug dependence	.02		
Total substance use disorders	.02		

No correlation is statistically significant (p<.05).

Two series of supplementary analyses were conducted. First, to further examine issues of sample stratification, the regression models above were extended to conduct tests of effect modification by the stratification factors sex, ethnicity, and family SES at birth. Specifically, models were fitted to allow the effects of each measure of stress/adversity on LTL to vary with levels of the stratification factor, and Wald chi square tests used to test the equivalence of effects across levels of the factor. Second, to examine the sensitivity of the conclusions to the scaling of LTL a further series of analyses were conducted in which LTL was analysed: as a log transformed measure to reduce heteroskedasticity; after eliminating outliers over 2 standard deviations above the mean; as a latent variable using the observed replicates as indicators of a latent factor; and as a dichotomous (short/long) variable where ‘short’ was defined on the basis of a series of arbitrary cut-points ranging from those who fell into the lowest decile, the lowest quartile or below the median on the distribution of LTL. A Bonferroni corrected p-value of .0024 was applied for all analyses to control for the possibility of Type I errors due to multiple significance testing.

## Results

### Associations between developmental factors (age 0–25) and LTL (age 28–30)

Table 1 reports the partial correlations and tests of significance between a series of 26 measures of life course stress or adversity assessed prior to the age of 25 years and LTL at age 28–30 adjusted for sex, ethnicity and family SES at birth. The predictors of telomere length are organised into a series of groups reflecting various developmental stages spanning: the antenatal/perinatal period; childhood; adolescence and young adulthood. The pervasive feature of the Table is a consistent lack of association between life course stress or adversity and LTL. In general the correlations were very small ranging from −0.06 to 0.06 with a median of 0.01, and none was statistically significant. This absence of association held for both specific measures and for the summary risk scores for each developmental domain.

### Sensitivity analysis

To examine the extent to which the findings in Table 1 held in general, further analyses were conducted to examine issues of sample stratification and variations in the measurement of telomeres (see statistical analysis).

### Sample stratification

A series of tests were conducted to examine the extent to which the associations between measures of stress/adversity and LTL varied with levels of the stratification factors sex, ethnicity, and SES at birth. In six of the 78 tests conducted there was evidence of significant (p<.05) differences in the strength of association across strata. However, only one of these (SES and number of mental health problems, p = .004) approached the Bonferroni corrected level of significance (p = .0024): among high SES (professional/managerial) families the association with LTL was negative (β = −.14, p<.01) compared to the associations in those from moderate or lower SES families (β = .08, .01 respectively, p>.10).

### Variations in the measurement of telomeres

There was no evidence that the findings of the analysis varied with the way in which telomere length was scaled or classified.

### General conclusions

When due allowance was made for multiple significance testing, irrespective of the ways in which the telomere data were analysed, life course stress or adversity up to the age of 25 was unrelated to LTL at age 28–30 in this well-studied birth cohort.

## Discussion

In this paper we have used data gathered over the course of a 30 year longitudinal study to examine the relationships between exposure to a wide range of life course stressors and telomere length at age 28–30 in a sample of 677 study participants who provided DNA and for whom satisfactory LTL measurements were obtained. The life course stressors spanned: pregnancy and perinatal events (birth weight, gestational age; pregnancy smoking) childhood circumstance (childhood maltreatment; family conflict) adolescent and young adult measures (substance use and misuse; mental health; life events). In total a series of 26 associations between LTL at age 28–30 were examined. The partial correlations adjusting for sex, ethnicity and family SES at birth ranged from −0.06 to 0.06 with a median value of 0.01, and in no case was there a significant association between life course stress and LTL. These findings were supported in further analyses examining the implications of stratification by sex, ethnicity or SES, and varying using different approaches to handling the LTL data. All analyses led to the common conclusion that when due allowance was made for multiple tests of significance there was no evidence from this cohort that a wide range of life course stressors assessed up to the age of 25 were related to LTL at 28–30 years.

These findings are inconsistent with several previous research reports [5], [53] about the effects of environmental stressors on telomere length. There are a number of possible explanations for these findings. The first explanation is that the measurement of LTL does not provide a sensitive measure of the effects of environmental stress and that a better measure is that of telomere erosion obtained by comparing telomere length before and after exposure to stress. While this explanation is possible it implies that LTL and telomere erosion are poorly correlated measures. The only circumstance under which this can occur is if initial telomere length is negatively correlated with subsequent telomere length. Further, LTL has been used in many previous studies as a proxy measure of telomere erosion [14], [19]–[26], [28], [29], [31]. Only one recent study used telomere erosion measures, in 236 children exposed to at least one violent incident, and concluded that violence exposure before age five can result in accelerated telomere erosion by age ten [27]. Notwithstanding these points, there is a clear need to replicate the findings of this study using measures of telomere erosion.

The second explanation is that LTL was inadequately assessed. This explanation does not seem likely since we have used well recognised standardised measures to assess LTL. Our T/S ratios were carefully controlled by inclusion of standard samples in each run, and the assay was validated by comparison of 20 control samples also subjected to the TRF method of telomere length measurement. This comparison yielded an r^2^ correlation of 0.71 (equivalent to r = 0.853, equivalent to r = 0.847 reported by Aviv and Blackburn [41]. In addition, our T/S ratio distribution and range is similar to that in other reports [4], [22], [23], [27].

The third explanation is that we have not assessed the types of stressor that have adverse effects on telomere length. This seems unlikely since we have assessed a comprehensive set of well recognised life course stressors. To our knowledge no previous study has examined the relationship between telomere length and life course stress using such a comprehensive set of measures. However, it is worth noting that there is considerable heterogeneity in the measures and study designs used for telomere length association analyses [54]–[57], and this may also impact on replicability of findings.

The final explanation is that during the period from birth to age 25, life course stressors do not have detectable effects on LTL. Several studies have reported an association between stress during childhood and shorter telomere length in adulthood [23]–[29]. Six of these studies were of a cross sectional nature, with retrospective collection of adversity data in all but one [28]. Four had small samples sizes and were relatively preliminary studies [23], [25], [26], [28]. The study of Kananen *et al*. (2010) was larger, involving subjects aged 30–87 with anxiety disorders and matched controls (total n = 939), and it found that the number of reported childhood adverse life events was associated with shorter telomere length in adults [24]. An even larger retrospective study on a population-based sample of women aged 41–80 years (n = 4441), reported suggestive evidence for association of adverse childhood experiences with shorter telomere length in adulthood [29]. Finally, a large retrospective study of UK twins (n = 1090) found no correlation between childhood maltreatment and telomere length. The diversity in approaches, sample sizes, age of participants, measures of stress, method of telomere length measurement [57], and the predominantly retrospective nature of these reports, means it is difficult to form confident conclusions from these studies.

In summary, the two most likely explanations of our findings are either: a) LTL is not a good measure of telomere erosion or b) that up to the age of 25 life course stressors have negligible effects on LTL and telomere erosion. Clarification of these alternatives will require data from further longitudinal research which has the capacity to measure telomere length prior to and following stress exposure.

## Supporting Information

Table S1 **Summary data for measures of life course stress and adversity.**
(DOCX)
