# Supplementary material for: No Association between Mean Telomere Length and Life Stress Observed in a 30 Year Birth Cohort
Source: PLoS One. 2014 May 9;9(5):e97102. doi: 10.1371/journal.pone.0097102 (PMC4016252; doi:10.1371/journal.pone.0097102)
Supplement: Table S1 — Summary data for measures of life course stress and adversity. (DOCX) [file pone.0097102.s001.docx]

**Table S1.** Summary data for measures of life course stress and adversity.

| Measure | Mean (SD)/% | Range | Cut-point Used to Define Adversity Indicator, % |
| --- | --- | --- | --- |
| Antenatal/Perinatal Factors |  |  |  |
| Birthweight (g) | 3358 (526) | 1270-4880 | <2500g, 5.0% |
| Gestation (wk) | 39.7 (1.6) | 29-44 | <36wk, 2.3% |
| Maternal smoking during pregnancy (cigs/day) | 4.1 (7.8) | 0-50 | ≥20 per day, 8.9% |
| Admission to neonatal intensive care | 17.4% |  |  |
| Antenatal/perinatal risk score | 0.34 (0.69) | 0-4 |  |
| Child Abuse/Family Violence (0-16 years) |  |  |  |
| Childhood sexual abuse | 0.35 (0.88) | 0-3 | Contact CSA, 12.3% |
| Childhood physical abuse | 1.17 (0.58) | 0-3 | Regular or severe punishment, 16.6% |
| Parental care score | 28.9 (6.2) | 2-36 | Lowest decile, 9.4% |
| Inter-parental violence and conflict | 9.2 (2.2) | 8-24 | Highest decile, 11.1% |
| Child abuse/family violence risk score | 0.49 (0.82) | 0-4 |  |
| Adolescent/Young Adult Substance Misuse (16-25 years) |  |  |  |
| Nicotine dependence | 31.4% |  |  |
| Alcohol dependence | 13.3% |  |  |
| Cannabis dependence | 13.8% |  |  |
| Other drug dependence | 4.1% |  |  |
| Number of substance use disorders | 0.63 (0.92) | 0-4 |  |
| Adolescent/Young Adult Mental Health (16-25 years) |  |  |  |
| Major depression | 42.9% |  |  |
| Anxiety disorder | 33.7% |  |  |
| Suicidal ideation/attempt | 28.7% |  |  |
| Traumatic life event score (lifetime) | 4.4 (3.0) | 0-18 | Highest quartile, 29.0% |
| Mental health problems score | 1.34 (1.28) | 0-4 |  |
| Adolescent/Young Adult Life Events (16-25 years) |  |  |  |
| Employment related events | 2.11 (0.98) | 0-3 |  |
| Serious illness/accident events | 1.88 (0.94) | 0-4 |  |
| Serious relationship problems | 2.01 (1.08) | 0-4 |  |
| Victimisation events | 1.06 (0.82) | 0-4 |  |
| Pregnancy/parenthood events | 0.57 (0.86) | 0-4 |  |
| Average total life events per annum | 2.13 (1.29) | 0-8 |  |
